# Piercing of the Lumbocostal Ligament by the Subcostal Nerve: A Previously Unreported Case

**DOI:** 10.7759/cureus.1825

**Published:** 2017-11-07

**Authors:** Marc Vetter, Joe Iwanaga, Rod J Oskouian, R. Shane Tubbs

**Affiliations:** 1 Seattle Science Foundation; 2 Swedish Neuroscience Institute; 3 Neurosurgery, Seattle Science Foundation

**Keywords:** anatomy, thoracic nerve, ribs, surgery, spinal fusion, variation

## Abstract

As lateral approaches gain popularity in lumbar spine surgery, detailed discussions regarding anatomical variations in the innervation of the thoracolumbar region are of increasing importance. Damage to intercostal or subcostal nerves can lead to post-operative complications including regional loss of sensitivity, motor function, or abdominal wall hernias. More specifically, the subcostal nerve has been identified in the literature as one of the more vulnerable structures during such procedures. A clear understanding of the position of the subcostal nerve relative to nearby anatomical structures is therefore important for medical professionals. We herein report a rare anatomical variation in which the subcostal nerve pierces the lumbocostal ligament.

## Introduction

The 12th thoracic ventral spinal rami, more commonly known as the subcostal nerve, lies inferior to the 12th rib. It is connected to a sympathetic trunk ganglion by both grey and white rami communicantes [1]. The subcostal nerve is the largest of the thoracic spinal ventral rami and often has a communicating branch with the first lumbar ventral ramus. After exiting the spinal column below the 12th thoracic vertebra, the subcostal nerve runs either anterior or inferior to the lumbocostal ligament. As it travels in a ventrolateral direction, the subcostal nerve passes inferior to the lateral arcuate ligament before piercing the transversus abdominis muscle, traveling between the transversus abdominis and the internal oblique muscle and innervating the external oblique muscle [2]. A large, lateral cutaneous branch of the subcostal nerve continues to the skin of the anterior gluteal region, in some cases descending as far as a few centimeters above the iliac crest [3-4].

## Case presentation

**Figure 1:**
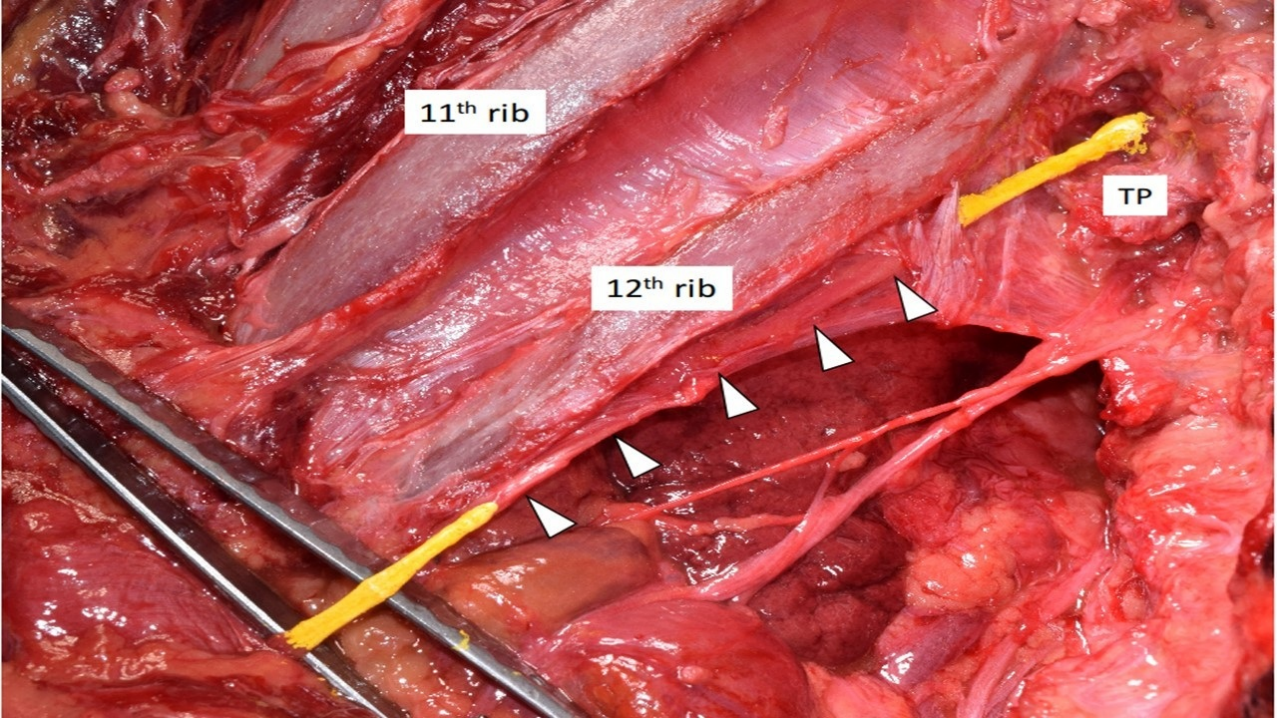
Cadaveric dissection of the left posterior thoracolumbar region at the T12/L1 level The subcostal nerve (colored yellow) is seen piercing the lumbocostal ligament (arrowheads). TP: transverse process of the first lumbar vertebra

The thoracolumbar region of an 81-year-old Caucasian male fresh frozen cadaver was dissected using a posterior approach. During dissection, a variation in the course of the subcostal nerve was observed on the left side. The subcostal nerve, emerging below the 12th thoracic vertebra slightly inferior to the 12th rib, pierced the lumbocostal ligament posteriorly (Figure 1). In this specimen, the lumbocostal ligament stretched between the left lateral edge of the first lumbar vertebra to the inferior edge of the 12th thoracic vertebra. Continuing laterally on the anterior side of the lumbocostal ligament, the subcostal nerve then emerged at the junction between the ligament and the rib. From this point, the nerve followed a typical anatomical course. The right subcostal nerve in this specimen did not pierce the lumbocostal ligament. 

## Discussion

The subcostal nerve is one of the most important and vulnerable nerves encountered in the thoracolumbar region [5-6]. In a study analyzing the branching of nerves in the anterolateral abdominal wall, Alonso et al. [5] found that on average, the subcostal nerve had eight separate branches – a greater number than both the 11th intercostal nerve and L1 nerve, superior and inferior to the subcostal nerve, respectively. Furthermore, the subcostal nerve regularly runs around 2 cm below the 12th rib [7], exposing it to iatrogenic damage during surgical procedures [6]. In contrast, the first through 11th intercostal nerves tend to run along grooves in the corresponding ribs, mitigating the risk of partial or complete transection [8]. The lumbocostal ligament may have an important role to play in preventing damage to the subcostal nerve. As noted by Saker et al. [8], due to its proximity, utilizing the lumbocostal ligament as a landmark during surgery may help surgeons to visualize and locate both the subcostal nerve and the adjacent dorsal rami as they exit below the 12th thoracic vertebra.

Saker et al. [8] also discuss the two common paths taken by the subcostal nerve relative to the lumbocostal ligament. They found that the subcostal nerve usually passes either anterior (9/20) or inferior (11/20) to the lumbocostal ligament. However, the present case did not fall into either category, as the lumbocostal ligament was pierced by the subcostal nerve. To our knowledge, this kind of case has not been documented in extant medical literature and provides an important example of a new anatomical variation. Additionally, one might speculate that such cases could represent a site of nerve entrapment or predispose a patient to symptoms of nerve entrapment at this location.

## Conclusions

Awareness of a broad range of anatomical variations is of critical importance for surgeons. The variation reported herein may help to better utilize the lumbocostal ligament as a landmark when locating the subcostal nerve posteriorly. Once identified, the course of this nerve to the anterolateral abdominal wall may be more easily ascertained. To our knowledge, this is the first report of such a variation in extant medical literature.
